# Population genetics of *Anopheles koliensis* through Papua New Guinea: New cryptic species and landscape topography effects on genetic connectivity

**DOI:** 10.1002/ece3.5792

**Published:** 2019-11-04

**Authors:** Luke Ambrose, Jeffrey O. Hanson, Cynthia Riginos, Weixin Xu, Sarah Fordyce, Robert D. Cooper, Nigel W. Beebe

**Affiliations:** ^1^ School of Biological Sciences University of Queensland Brisbane Qld Australia; ^2^ Department of Forensic Medicine University of Copenhagen Copenhagen Denmark; ^3^ ADF Malaria and Infectious Disease Institute Enoggera Qld Australia; ^4^ CSIRO St Lucia Qld Australia

**Keywords:** *Anopheles*, landscape genetics, malaria, microsatellites, population genetics, rDNA ITS2, southwest Pacific

## Abstract

New Guinea is a topographically and biogeographically complex region that supports unique endemic fauna. Studies describing the population connectivity of species through this region are scarce. We present a population and landscape genetic study on the endemic malaria‐transmitting mosquito, *Anopheles koliensis* (Owen). Using mitochondrial and nuclear sequence data, as well as microsatellites, we show the evidence of geographically discrete population structure within Papua New Guinea (PNG). We also confirm the existence of three rDNA ITS2 genotypes within this mosquito and assess reproductive isolation between individuals carrying different genotypes. Microsatellites reveal the clearest population structure and show four clear population units. Microsatellite markers also reveal probable reproductive isolation between sympatric populations in northern PNG with different ITS2 genotypes, suggesting that these populations may represent distinct cryptic species. Excluding individuals belonging to the newly identified putative cryptic species (ITS2 genotype 3), we modeled the genetic differences between *A. koliensis* populations through PNG as a function of terrain and find that dispersal is most likely along routes with low topographic relief. Overall, these results show that *A. koliensis* is made up of geographically and genetically discrete populations in Papua New Guinea with landscape topography being important in restricting dispersal.

## Graphical abstract

In a population genetic study of the malaria mosquito *Anopheles koliensis* through Papua New Guinea (PNG), we use ribosomal DNA genotyping, nuclear microsatellites, along with mitochondrial and nuclear DNA sequencing to reveal genetic and geographically district populations through PNG and one reproductively isolated cryptic species was identified in northern PNG. Using landscape genetic methods, we show that this geographic structure observes through PNG is best explained by landscape topography, with slope (or elevation) presenting as a significant factor.

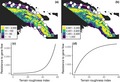

## 1 INTRODUCTION

### 1.1 Background

New Guinea is a large island in the southwest Pacific of unusual topographic, geological, and biogeographic complexity. Its western margin forms the boundary between the Oriental and Australian faunal regions. The complex geological makeup of New Guinea is due to it being the amalgamation of numerous geological units. The island was formed by the accretion of an Australian craton—the southern part of the island and numerous Pacific terranes and island arcs—forming the northern part of the island (Burrett, Duhid, Berry, & Varne, 1991; Pigram & Davies, 1987). Because of this complexity, New Guinea harbors a diverse array of locally endemic species (taxa that are endemic to certain parts of the island; Allison, 2007). Previously identified patterns of biological connectivity reveal that many New Guinean species show strong affinities to both Australia and Asia. The population structure and local endemicity observed in some species suggest that the geological history of the region has been important in the diversification of species. In many cases, vicariance is thought to play a more important role than dispersal in the current distributions of species in New Guinea and parts of Australasia (Heads, 2013). This theory is supported by the presence of biological breaks that occur in locations associated with historically separate landmasses in New Guinea as well as to the north and the south of the Central Range. This mountain range traverses the island from east to west, presenting a north–south barrier to dispersal for most species (Heads, 2013; Macqueen, Goldizen, Austin, & Seddon, 2011).

While the biogeography of New Guinea has been explored relatively extensively, there is less understood regarding the population genetics and phylogeography of individual species throughout the region. The Punctulatus Group of mosquitoes currently comprise 13 cryptic species that are widely distributed throughout New Guinea and northern Australia and show overlapping distributions. Some species of this group transmit malaria, and they provide a useful system with which to study the genetic diversity of organisms relative to the landscape features of this region. As larvae, they show highly specific aquatic requirements, however, as adults they can be on the wing and their ability to disperse is not well understood and may vary between species and sex (Ambrose et al., 2014). A study of two closely related isomorphic mosquito species in this group—*Anopheles hinesorum* Schmidt and the coastally restricted *Anopheles farauti* Laveran reveal similarities in the location of genetic breaks in New Guinea (Ambrose et al., 2012). In stark contrast, a third species in this group—*Anopheles punctulatus* showed little apparent population structure between these regions despite the use of fast‐evolving microsatellite markers (Seah, Ambrose, Cooper, & Beebe, 2013), suggesting it has undergone a recent range bottleneck and expansion throughout New Guinea and has recently traversed the Central Range.

Here, we focus on a fourth cryptic member and important malaria vector of the Punctulatus Group, *Anopheles koliensis* (Owen; Cooper et al., 2009; Slooff, 1964). This species is found throughout New Guinea, New Britain, and until recently on several islands of the Solomon Archipelago (Beebe & Cooper, 2002; Spencer, Spencer, & Venters, 1974; Taylor, 1975). In Papua New Guinea (PNG—eastern New Guinea), *A. koliensis* appears to be primarily a lowland species, inhabiting the river valley flood plains mostly below 300 m throughout the northwestern and southeastern lowland regions of PNG—it is rarely found in the distinct wet/dry monsoonal climate area of southwest PNG (Beebe, Russell, Burkot, & Cooper, 2015; Cooper & Frances, 2002). Surveys through PNG suggest oviposition occurs in natural ground pools and swamps, as well as in human‐disturbed or human‐modified sites, including vehicle wheel tracks and drains (Cooper, Waterson, Frances, Beebe, & Sweeney, 2002; Slooff, 1964). This mosquito was also found to have a positive association with human habitation (Cooper et al., 2002).

Due to both overlapping and variable adult morphology of the Punctulatus Group, molecular tools including genomic DNA probes and PCR are required for species identification (Beebe, Foley, Cooper, Bryan, & Saul, 1996; Beebe & Saul, 1995). The most utilized diagnostic tool is a PCR, restriction fragment length polymorphism analysis (RFLP) of the ribosomal DNA (rDNA) internal transcribed spacer 2 (ITS2). This diagnostic marker clearly discriminates *A. koliensis* from all other members in the Punctulatus Group based on agarose gel electrophoresis (Beebe & Saul, 1995).

An earlier study through northeast PNG (Madang and East Sepik region) using the PCR‐RFLP species diagnostic tool with a sensitive acrylamide gel size separation suggested *A. koliensis* comprised three ITS2‐RFLP variants (Benet et al., 2004). These ITS2 RFLP variants were designated genotype W, found only around Wosera in northeast PNG's Sepik region; genotype M, found only in the Madang region and MW, found throughout their study sites in northern PNG (Madang and Wosera regions). The authors suggested these genotypes may be evidence for distinct subspecies as some variation in time of night blood feeding was found (Benet et al., 2004). Variation in night feeding time may be important for the capacity of this species to develop behavioral resistance to the long‐lasting insecticide‐treated bed nets now deployed in the region (Russell, Beebe, Cooper, Lobo, & Burkot, 2013).

In this study, we further investigate the population genetics of *A. koliensis* in Papua New Guinea. We initially aim to verify the existence of the three previously described rDNA ITS2 RFLP variants within *A. koliensis* and assess reproductive isolation between individuals with these genotypes. We use additional nuclear loci (DNA sequence and microsatellite) as well as a mitochondrial locus to achieve this. We use this sequence and microsatellite data to describe the population genetic structure of *A. koliensis* throughout PNG and hypothesize that the complex geological history and landscape topography of New Guinea have shaped population structure in this mosquito. Finally, we use recently developed methods to assess whether landscape topography plays a role in maintaining this genetic structure, providing the first contribution to the literature on landscape genetics of a species in New Guinea.

## 2 MATERIALS AND METHODS

### 2.1 Mosquito collection, identification, and genotyping

**Figure 1:**
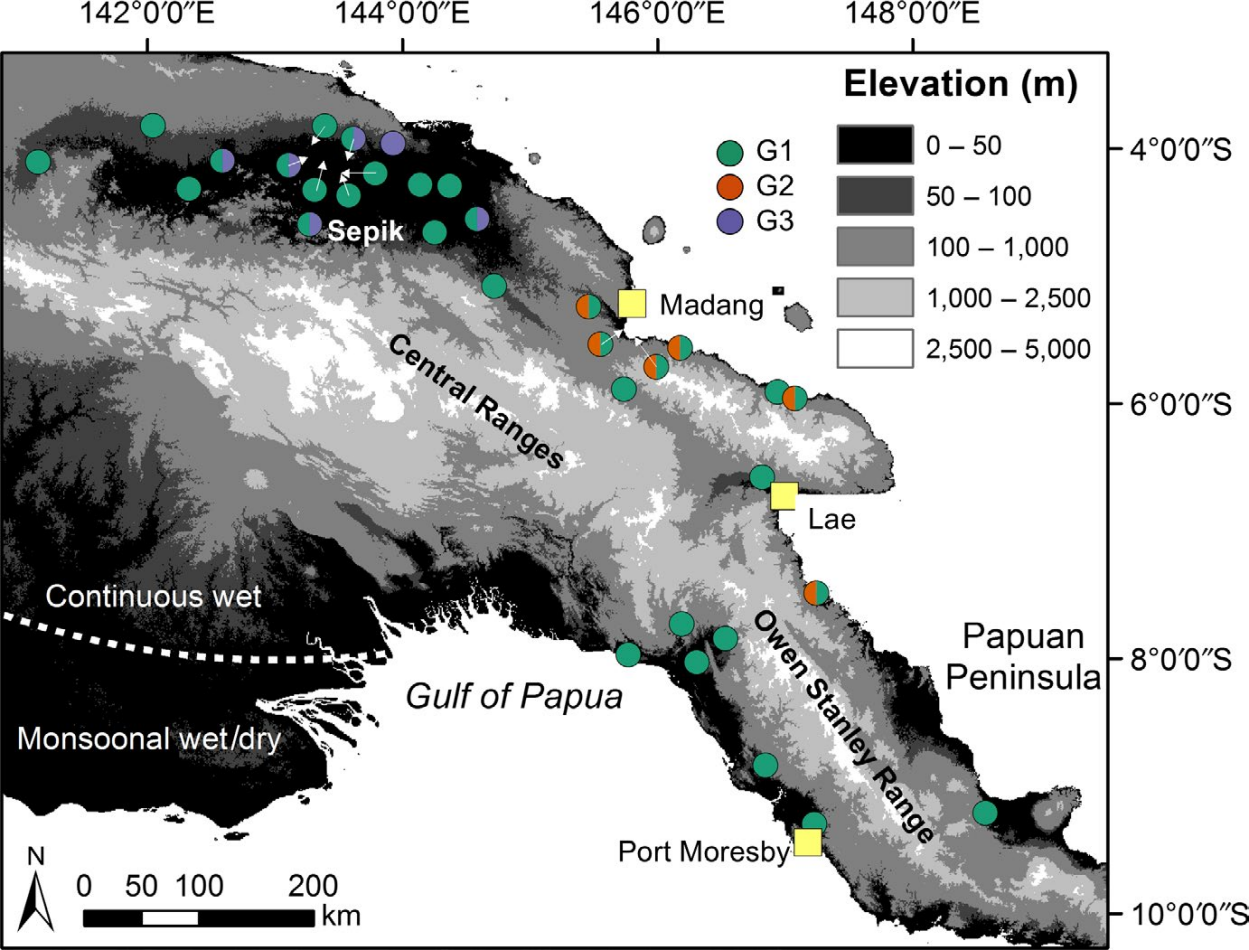
Topographic map of Papua New Guinea. Sites on map are *Anopheles koliensis* sampling sites used in this study (detailed in Table 1) and are color‐coded to show the distributions of the three rDNA ITS2‐RFLP genotypes identified

Mosquitoes were collected by human‐landing catches, CO_2_‐baited light traps, or through larval collections with the larvae being bred out to adults. Using these sampling techniques, *A. koliensis* was collected throughout PNG (Tables 1 and 2, Figure 1) and stored frozen, in alcohol, or desiccated on silica gel. Genomic DNA was extracted using a salt extraction method (Beebe, Ellis, Cooper, & Saul, 1999), and samples were identified to species by a species‐specific PCR‐RFLP of the ITS2 (Beebe & Saul, 1995). Only samples identified as *A. koliensis* were analyzed further. To confirm the presence of the M, MW, and W genotypes that were identified by using acrylamide gel electrophoresis RFLP separation (Benet et al., 2004), we used a 3% agarose gel and slightly longer ITS2 primers outlined in Alquezar, Hemmerter, Cooper, and Beebe (2010) for improved resolution of the three genotypes (Alquezar et al., 2010).

**Table 1:** Summary of the field sampling and rDNA ITS2 genotype classification

Site	Region	UIN	Year	Sample	*n* ITS2	ITS2^a^	*n* msats	Longitude	Latitude
1	Papuan	1754	1998	A	11	G1	11	−9.210437	148.571044
2	Gulf	1651	1998	A	18	G1	16	−7.840529	146.531037
3	Gulf	1654	1998	A	20	G1	20	−9.297003	147.229459
4	Gulf	1679	1998	A	6	G1	0	−8.837559	146.847221
5	Gulf	1664	1998	A	5	G1	0	−7.862963	146.391317
6	Gulf	1141	1994	A/L	9	G1	10, 6*	−7.969226	145.771503
7	Gulf	1170	1994	A	17	G1	9	−7.854744	146.339626
8	Madang/Lae	1425	1996	L	6	G1, G2(4)	6	−7.485226	147.246525
9	Madang/Lae	1383	1996	A	5	G1	0	−5.907663	146.940373
10	Madang/Lae	1403	1996	L/A/H	7	G1	7	−6.576148	146.820893
11	Madang/Lae	1365	1996	A	7	G1, G2(6)	0	−5.959224	147.078606
12	Madang/Lae	1321	1995	A	5	G1, G2(4)	5	−5.562424	146.176588
13	Madang/Lae	1330	1995	A	12	G1, G2(6)^b^	12	−5.239049	145.459759
14	Madang/Lae	1335	1995	L	16	G1, G2(7)	10	−5.477717	145.826484
15	Madang/Lae	1230	1995	H/A/L	25	G1, G2(7)	9	−5.414203	145.726434
16	Madang/Lae	1343	1995	A	14	G1	10	−4.55025	144.584708
17	Sepik	1236	1995	L/A	6	G1, G3(2)	0	−4.091523	143.563258
18	Sepik	1250	1995	A	16	G1, G3(4)	11	−4.187481	143.514957
19	Sepik	1384	1996	L	7	G1	0	−5.885432	145.738048
20	Sepik	1077	1993	A	6	G1	8	−3.819071	142.042905
21	Sepik	1081	1993	A	10	G1	7	−4.312503	142.326457
22	Sepik	1091	1993	A	9	G1	9	−4.103764	141.144154
23	Sepik	1109	1993	A	9	G1, G3(1)^b^	10	−4.093148	142.589981
24	Sepik	1116	1993	A	10	G1, G3(3)	5	−4.591389	143.274005
25	Sepik	1120	1993	A	7	G1	10	−4.191994	143.516772
26	Sepik	1136	1993	A	5	G3^b^	0	−3.956554	143.927244
27	Sepik	1118	1993	A	33	G1, G3(15)	31	−4.06985	143.256244
28	Sepik	1119	1993	A	10	G1	0	−4.092078	143.384137
29	Sepik	1131	1993	A	9	G1	0	−3.96853	143.282969
30	Sepik	1262	1995	A	5	G1	0	−4.282071	144.139967
31	Sepik	1348	1995	A	5	G1	0	−5.076785	144.718064
32	Sepik	1309	1995	A	6	G1	0	−4.653888	144.254696
33	Sepik	1266	1995	A	5	G1	0	−4.291936	144.37037

Abbreviations: A, adult; H, human‐landing catch; L, larval.

a Numbers of non‐G1 individuals found at that site are in parentheses.

b A subset of individuals from these sites were cloned and sequenced for the ITS2.

* Adult collection, larval collection.

**Table 2:** Population parameter summary for the mtDNA COI and nuDNA rpS9

Site	Region (site code)	UIN	*n* Rp9	Rp9 TD	Rp9 Pi	Rp9 Hd	*n* COI	COI TD	COI Pi	COI Hd
1	Papuan (CP144)	1754	9	−0.208	0.444	0.824	7	0.197	0.823	0.905
2	Gulf (CP16)	1651	6	1.183	1.046	0.848	10	−1.136	0.274	0.8
3	Gulf (CP20)	1654	7	1.274	1.024	0.89	10	−1.116	0.394	0.911
4	Gulf (CP51)	1679	5	0.024	0.58	0.356	5	−0.175	0.302	0.9
5	Gulf (GR7)	1664	4	1.238	0.796	0.679	5	−0.973	0.173	0.4
6	Gulf (GR3)	1141	4	1.098	1.029	0.75	3	NA	0.144	0.667
7	Gulf (GR59)	1170	1	NA	NA	NA	3	NA	0.432	0.667
8	Madang/Lae (LR106)	1425	4	NA	NA	NA	7	1.161	0.967	0.714
9	Madang/Lae (LR45)	1383	5	−0.038	0.380	0.778	5	−0.047	1.339	0.9
10	Madang/Lae (LR75)	1403	6	0.255	0.766	0.742	10	−1.507	0.504	0.867
11	Madang/Lae (LR9)	1365	5	0.024	0.58	0.622	8	−0.45	0.910	0.929
13	Madang/Lae (MP137)	1330	9	−1.173	0.249	0.608	9	−0.773	1.272	0.972
14	Madang/Lae (MP143)	1335	7	−0.634	0.642	0.824	10	−0.429	1.248	1
15	Madang/Lae (MP15)	1230	0	NA	NA	NA	4	NA	1.332	1
16	Madang/Lae (MP151)	1343	6	−0.205	1.458	0.924	8	−0.202	1.280	1
17	Sepik (MP21)	1236	0	NA	NA	NA	10	−1.525	1.085	1
18	Sepik (MP37)	1250	5	−0.22	1.280	0.978	10	−0.511	1.224	0.978
19	Sepik (MP47)	1384	3	0.520	0.779	0.8	2	NA	0	0
20	Sepik (SR22)	1077	0	NA	NA	NA	6	−1.445	0.792	1
21	Sepik (SR29)	1081	9	−0.941	1.387	0.863	7	−0.681	0.926	1
22	Sepik (SR41)	1091	7	−1.024	0.696	0.912	13	−0.733	1.329	0.974
24	Sepik (SR74)	1116	5	0.199	1.703	0.978	10	−0.973	1.641	0.978
26	Sepik (SR97)	1136	4	−0.075	1.135	0.929	5	0.0868	1.469	0.9

### 2.2 DNA sequencing and analysis of the ITS2, COI, and rpS9

Due to the multicopy nature of the rDNA, multiple variant copies (paralogues) of the ITS2 gene are often present within individual genomes and PCR products. These intragenomic sequence variants often cannot be directly sequenced using Sanger DNA sequencing as paralogues containing indels can collapse the chromatogram. For this reason, three individuals from sympatric populations from sites 1,330, 1,109, and 1,136 (see Table 1 for site localities) were PCR‐amplified for the ITS2 and cloned into bacteria with 3–5 randomly selected colonies sequenced following the methods of Alquezar et al. (2010).

A 527‐bp segment of the mtDNA COI and a 430‐bp region of nuclear ribosomal protein S9 (rpS9) were also amplified and directly sequenced following methods of Ambrose et al. (2012). For the rpS9, pseudohaplotypes of the nuclear locus were inferred using the program PHASE implemented in DNAsp v5 (Librado & Rozas, 2009). Additionally, DNAsp v5 was used to estimate haplotype diversity and nucleotide diversity, as well as to test for neutrality using Tajima's *D* (Tajima, 1989) and Fu's Fs (Fu, 1997) for each locus. Recombination was assessed using the program RPD3 (Martin et al., 2010). Haplotype networks were constructed using TCS 1.21 (Clement, Posada, & Crandall, 2000) under a 95% connection limit. Genetic diversity for mtDNA and rpS9 was estimated in a number of ways including haplotype diversity, haplotype richness and evenness (Kimura, 1964), and nucleotide diversity. Haplotype evenness provides an estimate of the number of equally frequent alleles that would result in the estimated haplotype diversity of the sampled population. Diversity estimates were obtained using the R packages gstudio (Dyer, 2014), pegas (Paradis, 2010), and adegenet (Jombart, 2008).

### 2.3 Microsatellite development and amplification

Primers for microsatellite analysis were obtained from 454 pyrosequencing data of the genomic DNA of *A. koliensis* following the methods of Ambrose et al. (2014). A total of 24 microsatellite markers were initially screened. Primers were selected based on their ability to consistently amplify a clean product (single locus) from all populations. No markers were excluded due to low variability, and details of primers used can be found in Table S1. Each locus was amplified by PCR using fluorescently labeled forward primers. The final PCR mixture contained 1× Taq buffer and 0.5 units of Taq (MyTaq; Bioline). The cycling involved an initial denaturation of 95°C for 3 min, and then 13 cycles of 95°C for 30 s, 56°C for 40 s with a decrease of 0.5°C/cycle, and 72°C for 30 s, followed by 25 cycles of 95°C for 30 s, 50°C for 40 s, and 72°C for 30 s, and a final 72°C for 5 min using minimum transition times. Amplified PCR products were genotyped by an external contractor, Macrogen.

### 2.4 Population parameters and statistics

Alleles for each marker were scored manually in the program GeneMarker (Holland & Parson, 2011). The presence of null alleles for each marker was checked at a population level (based on the populations found by subsequent analyses) using the program MICRO‐CHECKER (Van Oosterhout, Van Heuven, & Brakefield, 2004). Using these same population definitions, we checked for HWE, as well as calculating observed (Ho) and expected (He) heterozygosity in the program GenAlEx, v6 (Peakall & Smouse, 2006), and the program GenoDive (Meirmans & Van Tienderen, 2004) was used to calculate Fis for each population (Table S2 contains details of null alleles, HWE, etc.). FSTAT v2.9.3 (Goudet, 1995) was used to test for linkage disequilibrium between loci. To estimate population genetic structure between all pairs of sites (used subsequently for landscape genetic analyses), we used Hedrick's GST' (Hedrick, 2005), a measure which is not biased by within‐population variation as implemented in MMOD (Winter, 2012).

### 2.5 Population structure: Bayesian clustering

In order to assess broadscale population structure in New Guinea, we used a Bayesian clustering method as well as discriminant analysis of principal components (DAPC; Jombart, Devillard, & Balloux, 2010). The Bayesian clustering method was implemented in the program STRUCTURE v. 2.3.4 (Pritchard, Stephens, & Donnelly, 2000), which we ran through Structure_threader (Pina‐Martins, Silva, Fino, & Paulo, 2017). The admixture model with no location priors was used and run for 20 replicates of *K* [2, 8] for 500,000 generations with an additional burnin of 500,000 generations. We used the program CLUMPAK to assess the most likely *K* value and to produce final graphs based on the major mode of the best *K* (Kopelman, Mayzel, Jakobsson, Rosenberg, & Mayrose, 2015).

### 2.6 Population structure: multivariate approach (DAPC)

To complement the Bayesian analysis run in STRUCTURE (outlined below), we also performed a discriminant analysis of principal components (DAPCs) using the R package adegenet (Jombart, 2008; Jombart et al., 2010). This is a multivariate analysis designed for the use on genetic data such as microsatellites. It does not require that specific population genetic assumptions be met or try to fit data to a predefined model. Advantages over principal component analysis (and principal coordinate analysis) include that it is optimized to maximize variation among rather than within groups. We used the online server (Jombart, 2008) to determine the optimal number of principal components (PCs) to retain. We then performed DAPC on the five groups identified using STRUCTURE, retaining 60 PCs and 3 discriminant axes.

### 2.7 Landscape genetic analysis

We conducted a landscape genetic analysis to investigate the possible effects of landscape topography on genetic differentiation in *A. koliensis*. The most eastern population in the northern Papuan Peninsula in Figure 1 (site 1 in Table 1) was omitted from all landscape analyses due to this population consisting of a single collection site that showed a distinct site‐specific genetic signature. To describe topographic complexity across the study area, we obtained elevation data (90 × 90 m horizontal resolution) for PNG from the Shuttle Radar Topography Mission (SRTM; Jarvis, Reuter, Nelson, & Guevara, 2008) and used these elevation data to produce a layer describing landscape topography using the “terrain ruggedness index” (Wilson, O'Connell, Brown, Guinan, & Grehan, 2007). This index assigns greater values to areas that have higher (e.g., hills) or lower (e.g., valleys) elevation than their surroundings, and thus would be expected to positively correlate with dispersal cost (Row et al., 2015). After producing the terrain ruggedness layer at the 90 × 90 m resolution, the layer was then aggregated and resampled to a 5 × 5 km resolution to reduce computational burden. The landscape genetic analyses were conducted in R (version 3.4.1; R, 2011) and used the raster (Hijmans, 2014), rgdal (Bivand, Keitt, & Rowlingson, 2017), rgeos (Bivand & Rundel, 2014), and sp (Pebesma & Bivand, 2005) R packages for spatial data processing.

We assessed four different hypotheses for connectivity among the northern, southern, and combined populations. The first hypothesis was the null hypothesis, and under this hypothesis, pairwise measures of genetic variation were fit to a constant. The second hypothesis described the connectivity between a set of populations as a function of the geographic distance between them (Wright, 1943). The third hypothesis used terrain ruggedness to account for spatial variation in dispersal cost across the landscape and least‐cost path distances to model connectivity among populations. The fourth hypothesis also used terrain ruggedness and used commute distances to model connectivity among populations (Chandra, Raghavan, Ruzzo, Smolensky, & Tiwari, 1996). Thus, the third hypothesis and fourth hypothesis used different distance metrics to model connectivity. Least‐cost distances are best suited for modeling dispersal patterns that occur along the shortest and easiest to traverse routes (Adriaensen et al., 2003). Commute distances, on the other hand, are more suited for patterns that involve dispersal along multiple routes and are diffusive across the landscape. They are highly correlated with the more widely known “resistance distances” implemented in Circuitscape (Kivimäki, Shimbo, & Saerens, 2014; McRae, 2006), but are more computationally efficient. The least‐cost and commute distances were calculated using the gdistance R package (van Etten, 2015). To reduce computational burden, these distance metrics were calculated assuming movement in four directions.

Since landscape topography may be nonlinearly related with dispersal cost (Peterman, Connette, Semlitsch, & Eggert, 2014; Ruiz‐Lopez et al., 2016), we used genetic algorithms implemented in the ResistanceGA R package (Peterman, 2018) to identify the (near) best transformation for terrain ruggedness given different population sets (i.e., north, south, and combined) and connectivity metrics (i.e., least‐cost paths and commute distances). Briefly, these genetic algorithms were run using a population size of 250 and were terminated after they converged (i.e., no improvement after 200 consecutive iterations; using the ga R package; Scrucca, 2013). At each iteration in the algorithm, a candidate set of transformation parameters were generated, and the terrain ruggedness layer was transformed using the candidate set of parameters to generate a candidate dispersal cost layer. Next, pairwise distance measures were derived from the candidate layer, and the pairwise distance measures were fit to pairwise measures of genetic differentiation using linear mixed‐effects models (using the lme4 R package; Bates, Mächler, Bolker, & Walker, 2015). These models accommodated the nonindependence of pairwise values using a maximum‐likelihood populations (MPLE) parameterization (Clarke, Rothery, & Raybould, 2002). The log‐likelihood values associated with a given model, and in turn candidate cost or resistance surface, were used to assess the support for a given set of transformation parameters. Although the ResistanceGA R package offers both monomolecular and Ricker transformation functions (Peterman, 2018), we only examined monomolecular transformations because we expect terrain ruggedness to have a monotonic relationship with genetic differentiation. The pairwise least‐cost and commute distances measures derived from the best surfaces were subsequently used to examine the relative importance of surface topography on connectivity.

We used an information‐theoretic approach (Burnham & Anderson, 2003) to assess the relative support for each of the four connectivity hypotheses under the northern, southern, and combined populations. Linear mixed‐effects models were fit to the pairwise measures of genetic differentiation using the predictor variables for each connectivity hypothesis (as described above in the optimization process). The corrected Akaike information criterion (AICc) statistic was used to assess model performance due to low sample sizes (Burnham & Anderson, 2003). Following standard methodology (Burnham & Anderson, 2003), AICc statistics were calculated for each model, and the corresponding δAICc statistics and Akaike model weights (*w_i_*) were used to assess the relative support for each hypothesis (calculated using the MuMIn R package (Bartoń, 2017)). We also calculated marginal and conditional *R* ^2^ statistics to provide a more intuitive description of model fit (Nakagawa & Schielzeth, 2013). To assess uncertainty in the relative support for each hypothesis (following Dudaniec et al., 2016; Dudaniec et al., 2016), we conducted a bootstrap resampling analysis (10,000 replicates). In each bootstrap replicate, a subset of 75% of the populations in a population set (i.e., north, south, or combined) were randomly selected, the linear mixed‐effects models corresponding to each of the connectivity hypotheses (as described above) were refitted to the subset of populations, and the AICc statistics for the refitted models were calculated. After completing all of the iterations, we finally calculated the average rank and percentage of times that each hypothesis was found to have the most support among the bootstrap replicates.

## 3 RESULTS

### 3.1 Ribosomal DNA ITS2 genotype identification

**Figure 2:**
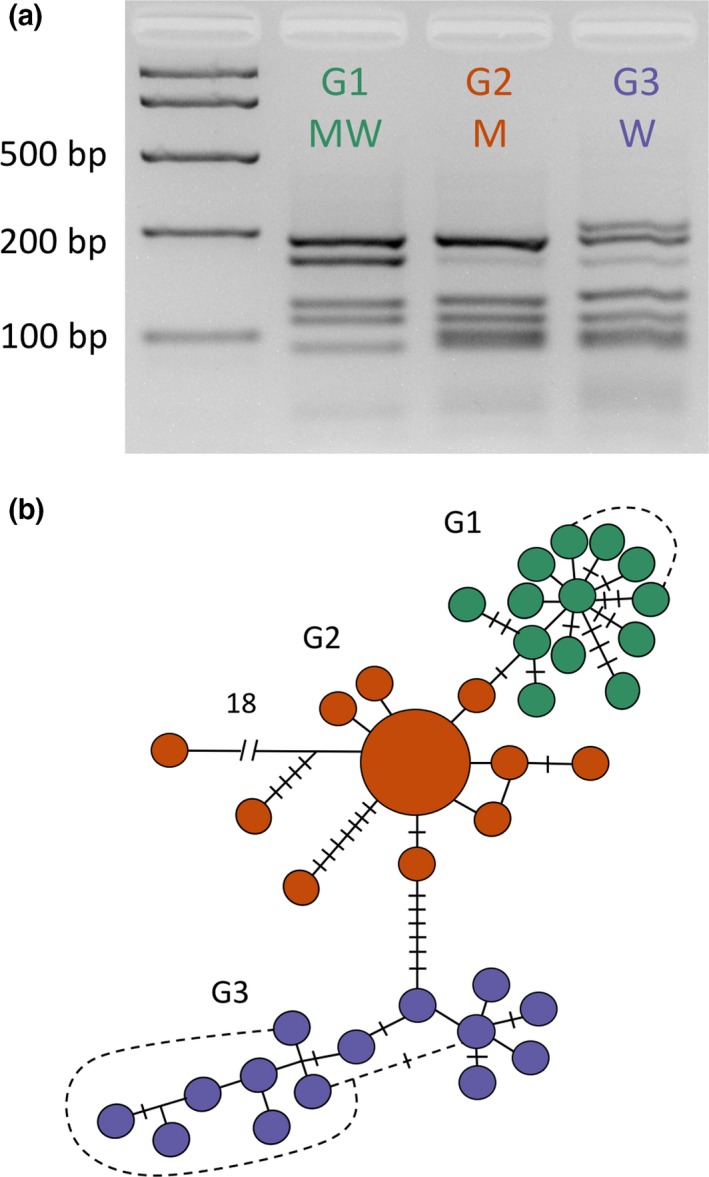
Three rDNA ITS2 restriction digest variants were found within *Anopheles koliensis* in PNG (G1, G2, and G3; upper panel). When a subset of these variants were cloned and sequenced, they revealed intraindividual paralogues and three distinct sequence variant lineages with no shared sequences between lineages. The haplotype network (lower panel) shows the genetic relationship of these cloned sequences with genotypes with G1 common through PNG with G2 and G3 showing nonoverlapping geographic restriction in northern PNG

All individuals included in this study were identified as *A. koliensis* by PCR‐RFLP of the ITS2 followed by restriction digest using *Msp* I (Beebe & Saul, 1995). Three subtle ITS2 restriction profile variants could be identified in the agarose gel that reflected those previously found using 10% acrylamide gels (Benet et al., 2004; see Figure 2). Of the 345 samples assessed from 33 sites throughout PNG, a common genotype termed G1 (equivalent to Madang/Wosera [MW] genotype in Benet et al., 2004) was identified from 32 collection sites. A second genotype (G2 equivalent to the Madang [M] genotype) was less common (five sites) and restricted to the Madang/Lae region of northern PNG. The third genotype (G3 equivalent to Wosera [W] genotype) was present at four sites (both inland and coastal) in the Sepik region only in northwest PNG. Thus, G2 and G3 appeared spatially structured in northern PNG—G2 in the eastern Sepik region and G3 in the Madang/Lae region. Only G1 was present south of the Central Range.

The ITS2 sequences were generated from plasmid clones drawn from a subset of individuals taken from sites where the genotypes could be regarded as being in sympatry in northern PNG (G1–G2 [site 13] and G1–G3); however, no sites were identified with all three genotypes present. As expected, ITS2 sequences clustered into three genetic groupings (see haplotype network in Figure 2b) that correlate with the three ITS2 genotypes identified by PCR‐RFLP analysis.

### 3.2 Population genetics: COI and rpS9 sequence analysis

**Figure 3:**
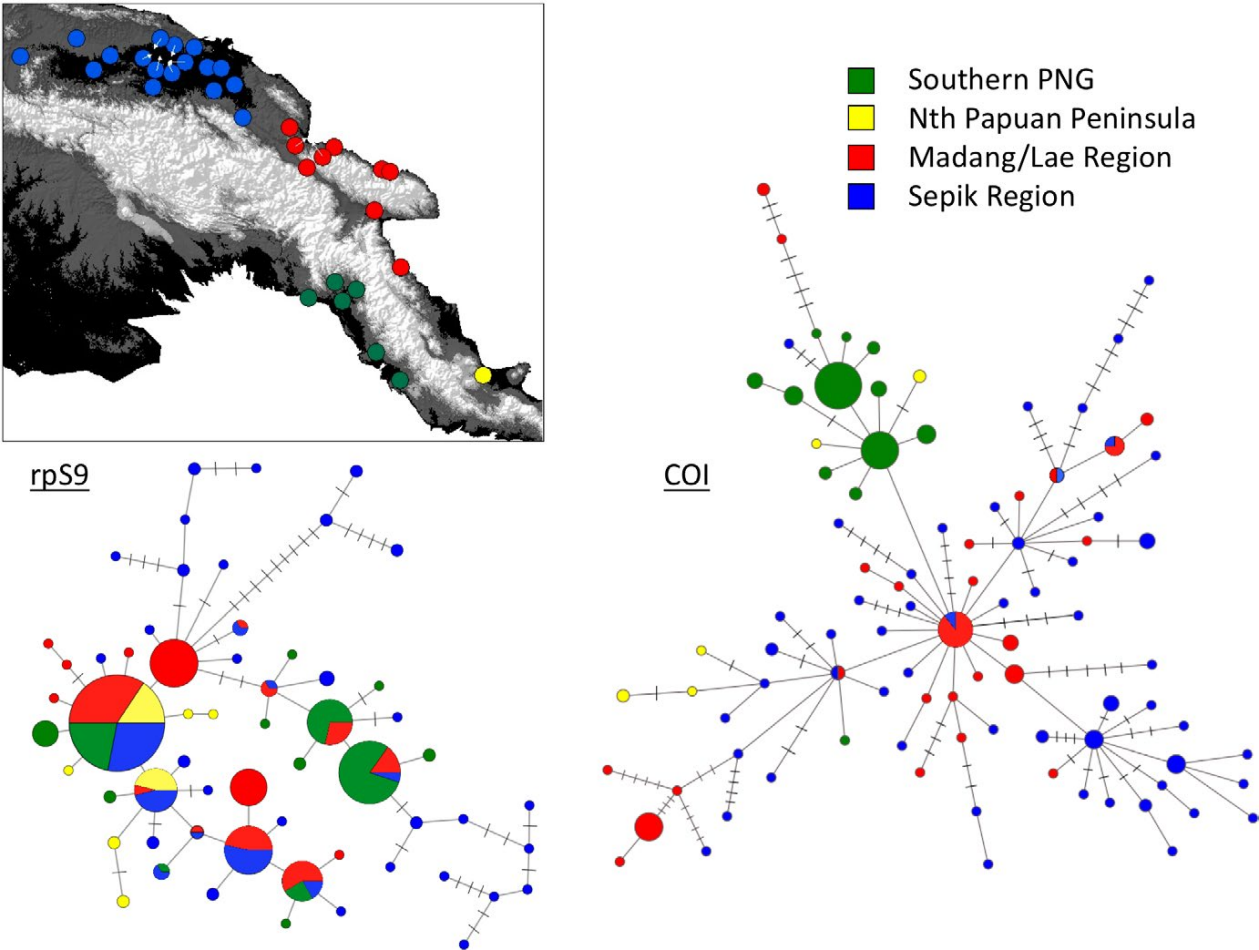
Haplotype network for the mtDNA COI and nuDNA rpS9 DNA sequences for *Anopheles koliensis*. The size of the circle reflects number of individuals sharing a particular sequence, and the connections represent single mutational steps. Colors designate regions in PNG were identified as genetically distinct by the microsatellites

The COI sequencing of 463 bp from 177 individuals revealed a high diversity with 99 unique haplotypes (haplotype diversity [Hd] = 0.984, nucleotide diversity/site [*π*] = 0.011). Neutrality tests using all sequences suggest an excess of low‐frequency polymorphisms present (significant test for Tajima's *D* (*p* < .05) of −2.01 and Fu and Li's *D* and *F* test statistic (*p* < .02) of −4.19 and −3.84, respectively. The phased 386‐bp‐long rpS9 sequence revealed 60 pseudohaplotypes from 107 individuals (Hd = 0.944, *π* = 0.011). Neutrality tests were mixed with a nonsignificant Tajima's *D*: (−1.56) but significant (*p* < .02) Fu and Li's *D* and *F* test statistic of −3.54 and −3.21, respectively. For both markers, these results indicate an excess of singletons. This is reflected in the haplotype networks for both loci which are displayed in Figure 3. Given the lack of common haplotypes, this is unlikely to be due to a recent population expansion or selective sweep and probably reflects a very large and stable population supporting very high diversity. Site‐specific spatial graphs showing mtDNA COI haplotype diversity (Hd) are presented in Figure S1.

The haplotype networks in Figure 3 suggest the rpS9 has more shared haplotypes between populations than the COI, and little discernible structure. Both mtDNA and nuclear loci show a high level of haplotype diversity with many unique haplotypes present in single individuals. At the rpS9 locus, a common haplotype was found in all geographic regions sampled, whereas at the COI locus there are shared haplotypes only between the two populations from northern PNG with these four haplotypes being shared between the Sepik region and Madang/Lae region.

### 3.3 Population genetics: microsatellite analysis

**Figure 4:**
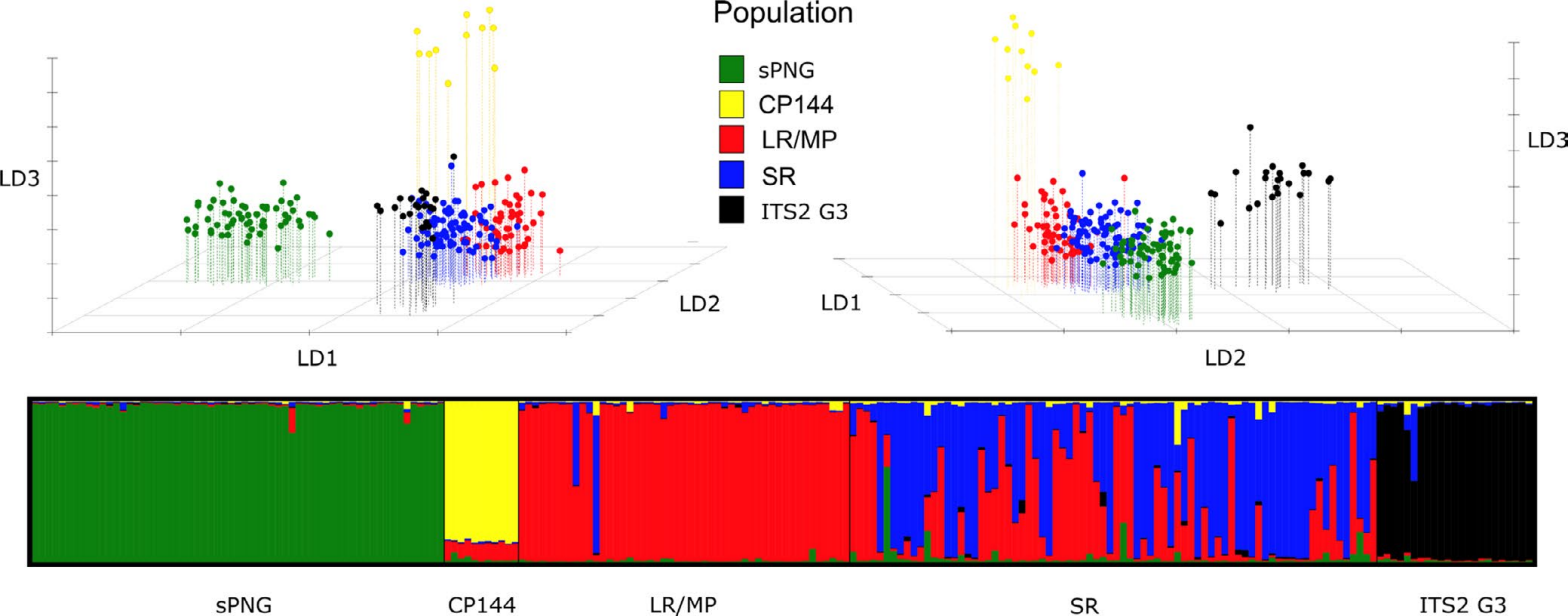
Analysis of population structure for *Anopheles koliensis* from PNG based on 11 microsatellite loci. The upper panel shows two views of a discriminant analysis of principal components (DAPCs). The lower panel shows results from the Bayesian STRUCTURE analysis (*K* = 5). Points and bars are colored to represent regional groups: sPNG = southern PNG (ITS2 G1), CP144 = northern Papuan Peninsula (ITS2 G1), LR/MP = Madang region (ITS2 G1 and G2), SR = Sepik region (G1), and ITS2 G3 = Sepik region (ITS2 G3)

Evidence of null alleles was found in some populations for some loci (Table S2). In STRUCTURE analyses, mean LnP(K) improved rapidly for *K* = 2 to *K* = 4 and plateaued at *K* = 5 and analyses run in CLUMPAK support *K* = 5 as the most likely *K* value. The barplot for *K* = 5 shows additional informative population structure over *K* = 4 and is presented in Figure 4. Two geographically defined populations can be clearly distinguished as separate genetic clusters, those being the southern New Guinean population and individuals from site CP144 (northern Papuan Peninsula). In addition to this geographically defined structure, individuals of ITS2 G3 (sampled from various sites in northern New Guinea in sympatry with other ITS2 genotypes) form another discrete group. This group may represent another cryptic species as there is no evidence of admixture with individuals of other ITS2 genotypes. Finally, there appears to be an east–west population break between the Madang/Lae region (sites 8–16) and the Sepik region (sites 17–33) with some evidence of admixture or incomplete sorting between these groups.

Groupings in the DAPC complement the STRUCTURE analysis and provide more detail into relationships between groups. Again, southern New Guinea, northern Papuan Peninsula, and individuals of ITS2 G3 form clear and separate groups. The Sepik population appears central in the DAPC cluster plot and may therefore have seeded all other populations. Individuals of the rDNA Genotype 3 appear to be most similar to individuals from the Sepik region (of other rDNA genotypes) with which they are sympatric. Again, the Sepik and Madang/Lae populations, while somewhat distinct, do overlap suggesting admixture or incomplete sorting. The southern New Guinean population appears most closely related to the Sepik population.

### 3.4 Effects of landscape topography on connectivity

**Figure 5:**
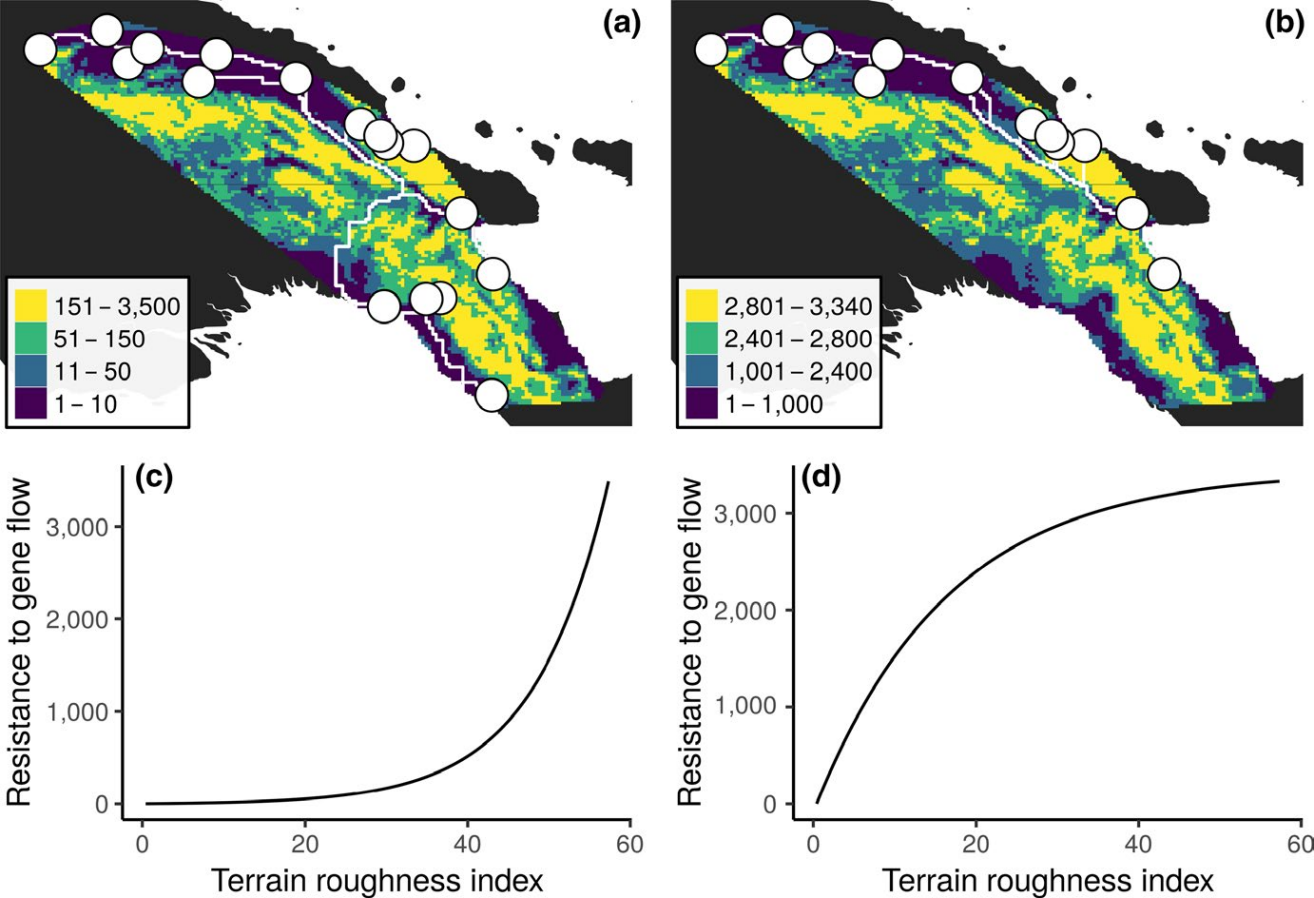
Landscape genetic analysis. The top panels show resistance to gene flow maps estimated using landscape topology for (a) all populations and (b) the northern populations. Colors show the spatial distribution of resistance to gene flow, points correspond to sampled populations, and lines represent potential dispersal routes between sampled populations using the resistance data. The bottom panels show the modeled relationship between landscape topology, measured as the terrain roughness index, and resistance to gene flow for (c) all populations and (d) the northern populations. Maps and data for the southern populations are not shown because the landscape genetic models fit exclusively to these populations failed to explain an adequate proportion of their genetic variation

Landscape topography had a large effect on connectivity among the *A. koliensis* populations at the broadscale (Figure 5a). The model that best explained genetic variation among all of the populations was the model that accommodated landscape topography and modeled dispersal using least‐cost path distances (Table 3). The relative support for this model eclipsed all others (*w_i_* = 1), and this model was able to explain a large proportion of the variation in genetic differences between populations (0.7*R* ^2^ *m*). This model also had the most support in 97.52% of the bootstrap replicates, showing that the support does not hinge on a specific combination of populations. Under this model, where surface topography was transformed using an inverse–reverse monomolecular function (Figure 5c, Table S3), lowland areas presented very little resistance to gene flow (shown in purple, blue, and green in Figure 5a) and it was only montane areas that presented substantial barriers to gene flow (shown in yellow Figure 5a).

**Table 3:** Effects of topology on connectivity among *Anopheles koliensis* populations

Populations	Connectivity model	δAICc	AICc	*w_i_*	*R* ^2^ *m*	*R* ^2^ *c*	Mean rank	Best model (%)
Combined	Topology and least‐cost distance	−328.61	0.00	1.00	0.70	0.75	1.03	97.52
	Topology and commute distance	−287.06	41.55	0.00	0.54	0.79	1.98	2.35
	Geographic distance	−206.14	122.47	0.00	0.04	0.47	3.12	0.13
	Null	−198.30	130.31	0.00	0.00	0.51	3.88	0.00
North	Topology and least‐cost distance	−201.99	0.00	0.99	0.43	0.56	1.55	70.93
	Topology and commute distance	−192.54	9.45	0.01	0.34	0.49	1.98	2.64
	Geographic distance	−188.98	13.01	0.00	0.16	0.41	2.54	26.43
	Null	−176.53	25.46	0.00	0.00	0.42	3.93	0.00
South	Null	−5.20	0.00	0.86	0.00	0.10	3.00	9.94
	Geographic distance	0.52	5.72	0.05	0.03	0.03	2.20	19.56
	Topology and commute distance	0.70	5.90	0.05	0.01	0.01	2.79	30.49
	Topology and least‐cost distance	0.75	5.95	0.04	0.01	0.01	2.00	40.01

Note

Data show results for maximum‐likelihood population effects (MLPE) describing genetic differences among combined, northern, and south populations. For a given model, AICc represents its corrected Akaike information criterion, δAICc is the difference between its AICc statistic and that of the best supported model in the set, and *w_i_* is its Akaike weight which denotes the probability that it is the best in the set. The marginal (*R* ^2^ *m*) and conditional (*R* ^2^ *c*) statistics are also reported. To account for uncertainty, the results from the bootstrap analysis are also reported. These show the mean rank of the models and the percentage of times that each model was the best in its set.

The connectivity between the northern populations was also affected by landscape topography (Figure 5b). Similar to the broadscale connectivity patterns, the best support connectivity model was the model that accommodated landscape topography between populations and described dispersal using least‐cost distances (*w_i_* = 0.99; Table 3). This model was able to explain an adequate proportion of the variation in genetic differences among the sampled populations (0.43*R* ^2^ *m*), and was also reasonably robust against uncertainty, having received the most support in 70.93% of the bootstrap replicates. Here, surface topography was transformed using a monomolecular function (Figure 5d, Table S3), where both highland (shown in green, Figure 5d) and montane areas (shown in yellow, Figure 5d) played a role in limiting gene flow between populations.

There was little evidence to suggest that landscape topography had any effect on connectivity among the southern *A. koliensis* populations. The best supported model was the null model (*w_i_* = 0.86; Table 3). However, the support for this model was not robust against uncertainty, with the null model only having the greatest amount of support in only 40.01% of the bootstrap replicates. One explanation for this result could be the presence of a couple of “outlier” populations which show markedly different genetic characteristics compared with other southern populations. For instance, when the connectivity models were fitted to a subset of populations that included the supposed outlier populations, none of the models might have been able to adequately explain the genetic differences among the populations, and so, the null model received the best support (observed in 9.94% of the bootstrap replicates). But when the connectivity models were fitted to a subset of populations that did not include the supposed outlier populations, the models were able to adequately describe the genetic differences among the populations, and so, the connectivity models which included landscape topography had much more support (e.g., terrain ruggedness with least‐cost distances had the greatest support in 40.01% of the bootstrap replicates).

## 4 DISCUSSION

The mosquito *A. koliensis* transmits malaria and is a member of a Punctulatus Group of 13 cryptic species of which 11 can still be distinguished by the original ITS2 PCR‐RFLP method (Beebe et al., 2015; Beebe & Saul, 1995). In assessing *A. koliensis* through PNG, we identified three ITS2 RFLP genotypes complementing a study on this mosquito in northwest PNG (Benet et al., 2004). A common genotype (G1 or Madang Wosera in Benet et al. (2004)) exists throughout PNG, with a second less common genotype (G2 or Madang in Benet et al.) sympatric with G1 through the Madang/Lae region. A third genotype (G3 or Wosera in Benet et al., 2004) was also sympatric with G1 but only through PNG's Sepik region in northwest PNG. Cloning and sequencing of individuals with these ITS2 genotypes revealed intraindividual ITS2 sequence paralogues but no shared sequences between individuals with different genotypes, despite genotypes occurring at sympatric collection sites (G1–G2 and G1–G3). The analysis of microsatellite data suggests that individuals of ITS2 genotype G3 may be reproductively isolated from individuals other with the other ITS2 genotype. Using landscape genetic analysis of microsatellite data, we found that elevation restricts the dispersal of *A. koliensis* between populations in Papua New Guinea.

### 4.1 Population structure of *A. koliensis* in PNG

Population structure is evident between north and south PNG in COI but not rpS9. The high haplotype diversity in both mtDNA COI and rpS9, and an excess of singleton sequences suggest that the species has a long history in the region. The higher effective number of haplotypes and *π* for the COI and rpS9 sequence data per site (Table 2) together suggest northern PNG may be an older and more stable population than southern PNG. We suggest that the northern New Guinean population may have founded the southern population incurring an apparent genetic bottleneck caused by the Central Range. The single population in eastern PNG, north of the Owen Stanley Range (site CP144), appeared genetically distinct at the level of the COI in that it did not share haplotypes with the other populations. However, haplotypes of individuals from this site appear in different parts of the haplotype network and the lack of haplotype sharing may be an artifact of the small sample size from CP144 (*n* = 11).

The analysis of the 11 microsatellites provided enhanced detail in regard to the spatial separation of populations through PNG. The population in southern PNG seen in the COI marker were now clearly identified, and the northern Papuan Peninsula population could be discriminated. Within northern PNG, microsatellites appeared to pull apart the Sepik region populations to the west from the Madang/Lae populations in the east—an area that could be regarded as continuous.

### 4.2 Effects of landscape topography on genetic connectivity

Overall, the broadscale differences among the *A. koliensis* populations could be better explained when considering the topography of the region as it is supported by both our model selection analysis and the bootstrap analysis for the combined set of populations. The least‐cost path model for mosquito dispersal suggests that movement between populations occurs along distinct routes, rather than a diffusive model for dispersal where individuals take multiple routes between each pair of populations. This phenomenon has also been observed in montane amphibian populations (Kershenbaum et al., 2014; Zancolli, Rodel, Steffan‐Dewenter, & Storfer, 2014).

Focusing only on the northern PNG populations, the best supported hypothesis for fine‐scale genetic variation among these populations also included topography with a least‐cost distance model for dispersal. This result suggests that at the fine scale, individuals in the northern populations may also exploit specific routes for dispersal. The bootstrap analyses corroborate this outcome, but they suggest that geographic distance may also play a minor role in gene flow. This is may be due to the limited number of northern populations that were sampled in the present study, and additional data covering more of the northern populations could potentially reduce this uncertainty.

Unlike the northern PNG populations, the best supported hypothesis for fine‐scale genetic variation among the southern populations was the null hypothesis. The relative support for the null hypothesis was far greater than any other hypothesis when fitting models to all of the sampled southern populations. However, the bootstrap analysis revealed that this strong level of support was only present when fitting models to all of the sampled southern populations and that the relative support for the null hypothesis was much lower when specific populations were omitted. Therefore, it seems likely that geographic distance and landscape topography have some effect on the spatial patterns of gene flow among southern populations. However, to understand the effect of landscape topography on gene flow among southern PNG *A. koliensis* populations, more extensive geographic sampling from this region is needed. These results further highlight the importance of quantifying uncertainty among competing hypotheses in landscape genetics (Dudaniec et al., 2016).

### 4.3 Reproductive isolation between sympatric rDNA variants and divergent biting behaviors in *Anopheles* mosquitoes

Microsatellite data suggest that individuals of ITS2 genotype G3 were reproductively isolated from individuals of the more common G1 and G2 ITS2 genotypes. Individuals of ITS2 genotype G3 appear to be spatially restricted to the western Sepik region of northern PNG, while individuals of ITS2 genotype G2 seem to be restricted to the Madang/Lae region, which supports the observed gene flow restriction between the Sepik and Madang/Lae populations. The ITS2, being part of the rDNA gene family, is tandemly arranged multicopy gene family in metazoans, and the evolutionary machinery maintaining sequence fidelity between copies in this array is not well described and does not follow traditional Mendelian rules of inheritance (Bower, Cooper, & Beebe, 2009; Eickbush & Eickbush, 2007; Nei & Rooney, 2005). In *Anopheles* mosquitoes, the rDNA is usually positioned on the sex chromosomes adjacent to the centromeres (Kumar & Rai, 1990) and the relatively rapidly evolving ITS2 spacer has been a useful marker for detecting early genetic discontinuity between populations (Alquezar et al., 2010; Beebe, 2018; Coleman, 2009; Muller, Philippi, Dandekar, Schultz, & Wolf, 2007). The positional effect of the rDNA being adjacent to the centromere would be to reduce recombination (Nachman & Churchill, 1996). In *Anopheles*, elevated levels of genetic divergence have been observed in regions proximal to the X chromosome in the face of gene flow across other parts of the genome (Reidenbach et al., 2012; Weetman, Wilding, Steen, Pinto, & Donnelly, 2012). If alleles for traits pertaining to time of night‐biting behavior occur near or within this low recombination landscape, the appearance of intraspecific behavioral differences may associate with rDNA divergence. Interestingly, the Benet et al. (2004) study observed variation in night‐biting behavior between the *A. koliensis* genotypes in northern New Guinea. Their study found evidence that the Madang (M) variant (G2 in this study) starts to blood feed later in the night, where MW (G1) and W (G3) were actively seeking a host as early as 6 p.m. It would be reasonable to hypothesize that genes for circadian or other rhythmic actives may be positioned near the centromere on the X chromosome.

Individuals of ITS2 genotype G3 are spatially restricted to the Sepik region of northern PNG and form a clearly separate group in the microsatellite clustering analyses, despite being sampled from the same sites as individuals of ITS2 RFLP genotype G1. The distinct microsatellite profile of these genotypes despite their overlapping ranges provides evidence that *A. koliensis* may well be more than one species in PNG. Individuals carrying the G1 and G2 genotypes show no evidence of genetic structure at other loci despite appearing to have different host feeding initiation times (Benet et al., 2004). We found that the geographic structure observed through PNG is best explained by landscape topography, with slope (or elevation) presenting as a significant factor. This makes sense given that the species exhibits a predominantly a lowland distribution (Cooper et al., 2002). Although the individuals of ITS2 G1 and G2 may have differences in time of feeding, they could not be separated by fast‐evolving microsatellites. Their distinct ITS2 genotypes may however represent early stages of genetic discontinuity which may, in time, lead to reproductive isolation across other parts of the genome.

## AUTHOR CONTRIBUTIONS

LA completed laboratory work and much of the genetic analyses and manuscript writing; JOH performed the landscape genetic analyses and wrote these sections in the manuscript; CR assisted with the genetic analyses and contributed to writing of the manuscript; WX performed a subset of the molecular genotyping, nDNA, and some mtDNA sequencing, while SLF initiated a substantial amount of the genotyping and mtDNA COI sequencing; RDC was responsible for the field collections; and NWB designed the project, contributed to field collections, and assisted in developing the manuscript.

## Data Availability

Microsatellites STRUCTURE text file available from Dryad https://datadryad.org/review?doi=doi:10.5061/dryad.qr2np73. DNA sequences: GenBank (COI: KU662130–KU662306, rpS9: MH999139–MH999278, and ITS2: KU139075–KU139117).
