# Supplementary figures and images for: Population genetics of Anopheles koliensis through Papua New Guinea: New cryptic species and landscape topography effects on genetic connectivity
Source: Ecol Evol. 2019 Nov 4;9(23):13375–88. doi: 10.1002/ece3.5792 (PMC6912914; doi:10.1002/ece3.5792)

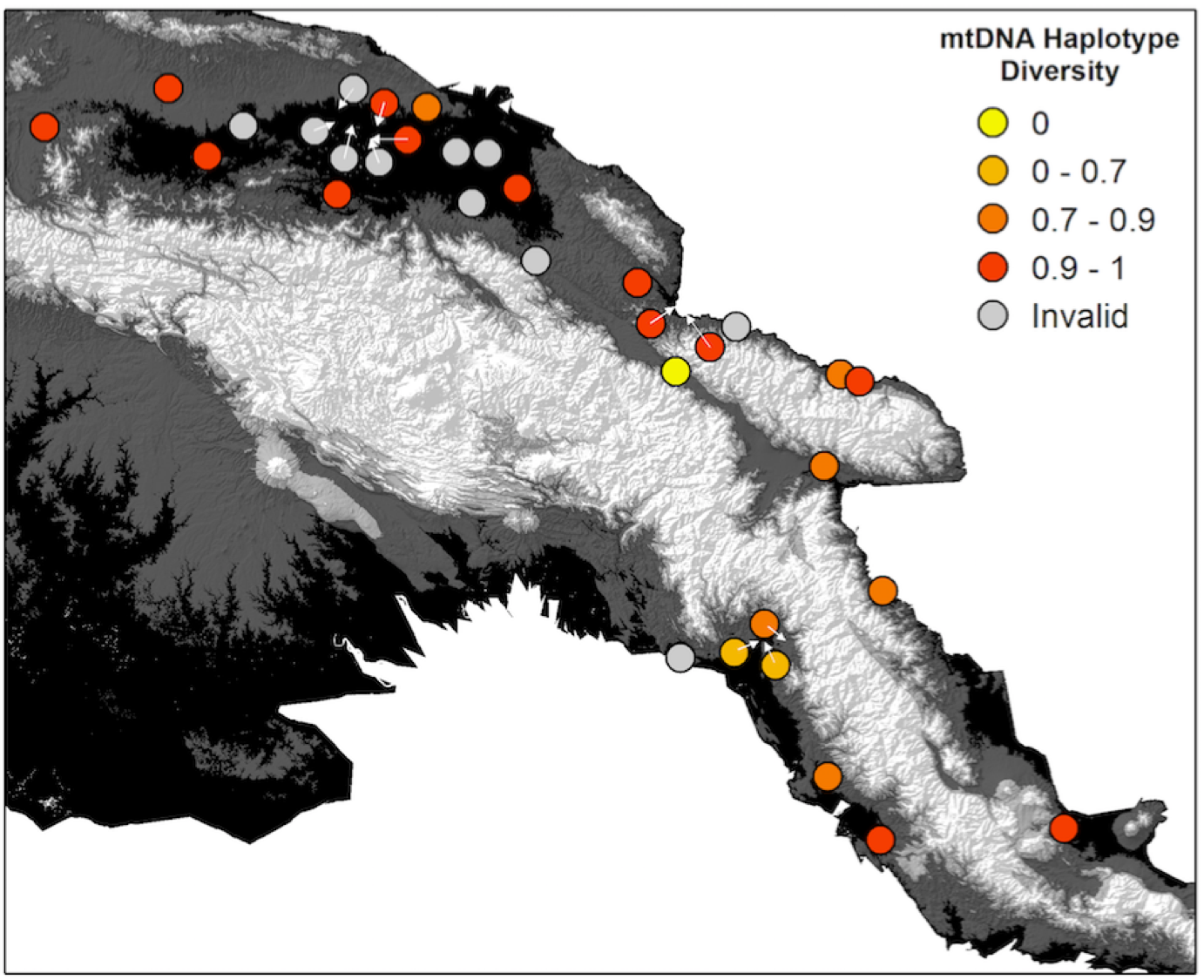

Supplement: Supplementary file 1 [file ECE3-9-13375-s001.png]
