# Supplementary material for: Population genetics of Anopheles koliensis through Papua New Guinea: New cryptic species and landscape topography effects on genetic connectivity
Source: Ecol Evol. 2019 Nov 4;9(23):13375–88. doi: 10.1002/ece3.5792 (PMC6912914; doi:10.1002/ece3.5792)
Supplement: Supplementary file 2 [file ECE3-9-13375-s002.docx]

**Table S1. Primer sets for the microsatellites, rpS9 and COI**

| **Marker** | **F primer** | **R primer** |  | **Size range** | **N alleles** |
| --- | --- | --- | --- | --- | --- |
| **kolDi-1** | CCCTCCATGAATACACGTATCG | CCATCGAGCAGTAGTACAGC |  | 139 - 199 | 29 |
| **kolDi-3** | CACCGGTAGCTTGTTGTTGC | GTATCGCTGGGACACATGTTC |  | 121 – 139 | 9 |
| **kolDi-6** | CATACGGTAGCGCGCATTAG | GCCAGACAAAGAGCTGATCG |  | 315 - 371 | 26 |
| **kolDi-9** | GATGCAGGCGGGTTAAGAAC | CGGGCGAAATTGACAACGAC |  | 339 – 387 | 18 |
| **kolDi-10** | ATCGATCGTTAATGCGCTGC | GCCGATGTAGCAATCGATGG |  | 126 – 188 | 26 |
| **kolTri-1** | AACCGACGCAACCATTCATC | GCAGGACCACGACAACTTAC |  | 363 – 408 | 15 |
| **kolTri-7** | GTGTAACCCAAAGCACCGAC | CCGAGCAGAAATCAGCAGAC |  | 158 – 194 | 16 |
| **kolTri-8** | TTCTGTTGCTTTCACCCTGC | AGAACCTGGAGATGCTGGTG |  | 168 – 195 | 9 |
| **kolTri-11** | GTCAGGACACCGTGCAAAC | GGTTCCTTCGCGCATACTTC |  | 321 – 402 | 26 |
| **kolTri-19** | TGGGTGTTGATCGGTGAGAC | GTACAGATGACGTGGCATCG |  | 180 – 207 | 8 |
| **kolTri-20** | CGCGATTGTGCTCTCTGTG | CGCACCATCCCATCAAAGTC |  | 180 – 219 | 14 |
| **COIf-250*** | GTTCCTTTAATATTAGGAGCACC | TAATAT AGCATAAATTATTCC |  | 527 | 99 |
| **rpS9*** | GAAAAGCCRCGTCTCGATGCGG | GCCAATCCCAGCTTGAASACC |  | 431 | 60 |

*From Ambrose *et. al.* (2012)
