# Supplementary material for: Population genetics of Anopheles koliensis through Papua New Guinea: New cryptic species and landscape topography effects on genetic connectivity
Source: Ecol Evol. 2019 Nov 4;9(23):13375–88. doi: 10.1002/ece3.5792 (PMC6912914; doi:10.1002/ece3.5792)
Supplement: Supplementary file 3 [file ECE3-9-13375-s003.docx]

**Table S2. Population parameter summary for the 11 microsatellites**

| Marker | Null Freq (Br) | Ho | He | Pops not in HWE | Null alleles |
| --- | --- | --- | --- | --- | --- |
| kolDi-1 | 0.182 | 0.913 | 0.618 | SR | SR, G3 |
| kolDi-3 | 0.067 | 0.498 | 0.405 | - | G3 |
| kolDi-6 | 0.105 | 0.891 | 0.711 | - | sPNG, LR/MP, SR |
| kolDi-9 | 0.047 | 0.819 | 0.738 | - | SR |
| kolDi-10 | 0.024 | 0.752 | 0.711 | - | - |
| kolTri-1 | 0.171 | 0.857 | 0.585 | sPNG, SR, G3 | sPNG, LR/MP, SR, G3 |
| kolTri-7 | 0.028 | 0.833 | 0.782 | SR | - |
| kolTri-8 | 0.066 | 0.764 | 0.656 | - | - |
| kolTri-11 | 0.423 | 0.919 | 0.348 | sPNG, LR, SR, G3 | sPNG, LR/MP, SR, G3 |
| kolTri-19 | 0.031 | 0.708 | 0.658 | - | - |
| kolTri-20 | 0.065 | 0.799 | 0.690 | - | - |
