# Supplementary material for: Population genetics of Anopheles koliensis through Papua New Guinea: New cryptic species and landscape topography effects on genetic connectivity
Source: Ecol Evol. 2019 Nov 4;9(23):13375–88. doi: 10.1002/ece3.5792 (PMC6912914; doi:10.1002/ece3.5792)
Supplement: Supplementary file 4 [file ECE3-9-13375-s004.docx]

**Table S3.** The resistance layer transformations used to test evaluate different models for connectivity among the *A. koliensis* populations. Data shows the name of the transformation, the shape, and maximum value parameters used to generate the near-optimal resistance surface for each hypothesis. The equation IRM denotes the inverse-reverse monomolecular transformation.

| **Connectivity model** |  | **Resistance transformation** | | |  | **Populations** |
| --- | --- | --- | --- | --- | --- | --- |
|  |  | **Equation** | **Shape** | **Maximum** |  |  |
| slope + LCP |  | IRM | 4.53 | 2495.7 |  | all |
| slope + RD |  | Ricker | 0.24 | 4.47 |  |  |
| slope + LCP |  | IRM | 1.7 | 1113.3 |  | northern |
| slope + RD |  | Ricker | 0.02 | 1166.33 |  |  |
| slope + LCP |  | IRM | 0.06 | 2410.77 |  | southern |
| slope + RD |  | IRM | 0.03 | 2499.64 |  |  |
